# The Protecting Effect of Deoxyschisandrin and Schisandrin B on HaCaT Cells against UVB-Induced Damage

**DOI:** 10.1371/journal.pone.0127177

**Published:** 2015-05-15

**Authors:** Wei Hou, Wei Gao, Datao Wang, Qingxiu Liu, Siwen Zheng, Yingping Wang

**Affiliations:** Institute of Special Animal and Plant Sciences, Chinese Academy of Agricultural Sciences, Changchun, China; Jilin University, CHINA

## Abstract

*Schisandra chinensis* is a traditional Chinese medicine that has multiple biological activities, including antioxidant, anticancer, tonic, and anti-aging effects. Deoxyschisandrin (SA) and schisandrin B (SB), the two major lignans isolated from *S*. *chinensis*, exert high antioxidant activities *in vitro* and *in vivo* by scavenging free radicals, such as reactive oxygen species (ROS). Ultraviolet B-ray (UVB) radiation induces the production of ROS and DNA damage, which eventually leads to cell death by apoptosis. However, it is unknown whether SA or SB protects cells against UVB-induced cellular DNA damage. Our study showed that both SA and SB effectively protected HaCaT cells from UVB-induced cell death by antagonizing UVB-mediated production of ROS and induction of DNA damage. Our results showed that both SA and SB significantly prevented UVB-induced loss of cell viability using 3-(4, 5-dimethylthiazol-2-yl)-2, 5-diphenyltetrazolium bromide (MTT) assays. Dichloro-dihydro-fluorescein diacetate (DCFH-DA) assays showed that the production of ROS following UVB exposure was inhibited by treatment with SA and SB. Moreover, SA and SB decreased the UVB-induced DNA damage in HaCaT cells by comet assays. In addition, SA and SB also prevented UVB-induced cell apoptosis and the cleavage of caspase-3, caspase-8 and caspase-9. In a word, our results imply that the antioxidants SA and SB could protect cells from UVB-induced cell damage via scavenging ROS.

## Introduction

The skin is repeatedly exposed to chronic ultraviolet (UV) irradiation, which induces various cellular responses, such as inflammation, aging, and even skin cancers [1–3]. It is well known that the protection from chronic UV irradiation is important to prevent skin cancer. Solar UV radiation is comprised of approximately 90–98% ultraviolet A (UVA), wavelength 320–400 nm, and 1–10% ultraviolet B (UVB), 290–320 nm. UVB, even being a minor component of sunlight UVB, reaching the earth surface, was found to be the most effective to induce skin cancer via experimental studies [1,4]. UV radiation usually produces small amount of Reactive Oxygen Species (ROS) and these ROS are magnified in a Ca^2+^-dependent manner by mitochondria, generating more ROS which can inhibit multiple PTPase activities. Inhibition of PTPases leads to a derepression of tyrosine kinases and activation of downstream signal pathways [5]. UV induces DNA damage through nucleotide excision, while excessive DNA damage beyond the intracellular repair capacity leads to the DNA damage response, which in turn promotes cell death by apoptosis [6]. Meanwhile, ROS is also an important intracellular DNA damaging agent that especially induces the oxidation of deoxyribonucleotide bases, leading to gene mutations. The direct DNA damage and ROS production caused by UV lead to the activation of various cell signaling pathways, which coordinately determine the death or survival of a cell following UV irradiation [6,7].

During evolution, each organism has been endowed with a complicated antioxidant system to neutralize ROS for survival [4,8]. Genetic mutations triggered by ROS resulting from UV irradiation are the primary cause of skin cancer. In addition, antioxidants could prevent UV-induced cancer. Some botanical ingredients are natural antioxidants that can effectively prevent UV-induced cellular damage and have few side effects. For example, persimmon leaf extract has a potential effect to prevent from UVB-induced skin inflammation [9], and tea polyphenols protect against UVA-induced cytotoxicity via apoptosis and inhibit lipid peroxidation [10].

*Schisandra chinensis* (*S*. *chinensis*) has antioxidant, anticancer, tonic, and anti-aging activities, which is widely used as a traditional Chinese medicine [11]. Recent studies showed that *S*. *chinensis* is a promising agent for the treatment of metabolic disturbances and oxygen free radical injury, such as inflammation, radiation injury, and reperfusion of ischemic organs [11]. The pharmacology and chemistry effects of *S*. *chinensis* have been extensively studied [11]. The most important active constituents of *S*. *chinensis* are lignans, including schisandrol A, schisandrol B, deoxyschisandrin (SA), and schisandrin B (SB), which have a dibenzocyclooctadiene skeleton [12]. *Schisandra sphenanthera*, in the same Schisandraceae family with *S*. *chinensis*, contains a variety of pharmacologically active lignans. It could reduce the expression of COX-2 and the PGE2 production induced by UVB in HaCaT keratinocytes [13]

Several studies showed that SB, a major lignan isolated from *S*. *chinensis*, has a high antioxidant potential both *in vitro* and *in vivo* [14,15]. Meanwhile, SB could protect skin from photo-aging with principle its pro-oxidant effect and the glutathione antioxidant response subsequently [16]. Similarly, SA has been demonstrated to have antioxidant activity and can inhibit H_2_O_2_-induced cell apoptosis [17,18].

UV radiation generate large amounts of ROS by mitochondria. The mitochondrial membrane potential was lost and the mitochondrial apoptotic proteins were released [19]. Caspase is eventually activated, contributing to induction of apoptosis[20]. In this study, We hypothesize SA and SB can scavenge ROS, decrease the DNA damage, increase the mitochondrial membrane protein activation and block caspase activation. Therefore, the present study was aimed to investigate the protective effects of SA and SB against UVB-induced damage in HaCaT cells.

## Materials and Methods

### Materials

Fetal bovine serum (FBS) and RPMI 1640 medium were purchased from Clark (Richmond, NC, USA), Life Technologies (Grand Island, NY, USA); penicillin, streptomycin, and 3-(4,5-dimethylthiazol-2-yl)-2,5-diphenyltetrazolium bromide (MTT) were bought from Sigma (St. Louis, MO, USA); the Annexin-V-Fluos Staining Kit was bought from Roche (Mannheim, Germany); the OxiSelect Comet Assay Kit was obtained from Cell Biolabs (San Diego, CA, USA); and the Pierce BCA Protein Assay Kit was acquired from Thermo Scientific (USA). The antibody against β-actin was obtained from Proteintech (Chicago, IL, USA). Caspase-8 and caspase-3 antibodies were received from Cell Signaling Technology (Boston, MA, USA). Active caspase-3 and caspase-9 antibodies were received from Abcam (Cambridge, UK). The horseradish peroxidase-conjugated secondary antibody (goat anti-rabbit) was obtainedfrom Proteintech (Chicago, IL, USA). SA and SB were purchased from the Control of Pharmaceutical and Biological Products (Beijing, China). DCFH-DA Reactive Oxygen Species Assay Kit and RIPA lysis buffer were gained from Beyotime (Haimen, China).

### Cells and cell culture

Human keratinocyte HaCaT cells were received from the American Type Culture Collection (ATCC, Rockville, MD). Cells were cultured in RPMI 1640 medium added with 10% FBS, 1% penicillin, and 1% streptomycin at 37°C 5% CO_2_.

### UVB irradiation

HaCaT cells were plated on culture dishes. The cells were treated with irradiation, covered with a thin layer of PBS after washed with phosphate-buffered saline (PBS), and exposed to UVB (30 mJ/cm^2^, 280–320 nm) from a bank of lamps (Spectronics Corp., Westbury, NY, USA) placed 25 cm. The irradiance of the lamps was calculated by a calibrated photometer (Spectronics Corp., Westbury, NY, USA), the cells supplied with fresh culture medium after exposure to UVB were incubated for the indicated times.

### MTT assay

HaCaT cells were plated into 96-well plates at 1.5 × 10^4^ cells/100 μL. When reached 80% confluence, the cells were added with 100 μM SA or SB for 24 h, followed by exposure to 30 mJ/cm^2^ of UVB. MTT solution (5 mg/mL) was added after 24 h, followed by incubation for an additional 5h. Then the medium was removed, and 150 μL dimethyl sulfoxide was supplied to dissolve formazan. A microplate reader measured absorbance at 540 nm. The ratio of optical density at 540 nm of each reagent-treated well to the corresponding control was calculated as a percentage.

### Measurement of ROS

The ROS level was evaluated by DCFH-DA assays. HaCaT cells were clutured into 6-well plates at 1.2 × 10^6^ cells/well. When reached 80% confluence, the cells were added with 100 μM SA or SB for 24 h, followed by exposure to 30 mJ/cm^2^ of UVB, the expression of intracellular ROS was performed by loading the cells with DCFH-DA (10 μM) for 20 min at 37°C after 24 h using fluorometry. The cells washed three times with fresh culture medium were placed on a laser scanning confocal microscope (Nikon, Tokyo, Japan). Fluorescence was detected using a 488 nm excitation and a525 nm emission.

### Comet assay

Comet assays were used to measure DNA damage in individual cells. HaCaT cells were plated into 6-well plates at 1.2 × 10^6^ cells/well, and when reached 80% confluence, the cells were treated with 100 μM SA or SB for 24 h, followed by exposure to 30 mJ/cm^2^ of UVB., The cells were gently removed and centrifuged at 700 × *g* for 2 min after culturing for an additional 24 h. The cells was washed with ice-cold PBS, added to Comet Agarose (1:10 ratio, v/v), and the mixture was transferred onto the OxiSelect Comet Slide at once. The slide was placed at 4°C for 15 min in the dark and then soaked in cell lysis buffer at 4°C for 60 min in the dark. Lysis buffer was sucked and replaced with pre-chilled alkaline solution at 4°C for 30 min in the dark. The slide was placed in a horizontal electrophoresis chamber filled with cold Tris/borate/EDTA electrophoresis solution and applied with 30 V for 15 min. After three washes with prechilled H_2_O and one wash with cold 70% ethanol for 5 min, the slide was placed to dry at room temperature. Once the agarose and slide were completely dry, 100 μL diluted Vista Green DNA Dye was added to the slide. Then the slide was incubated at 25°C for 15 min. The slide was observed under epifluorescence microscopy by a FITC filter (Lycra, Germany). The Comet analysis program used was CASP.

### Flow cytometric analysis

The treated cells were washed with ice-cold PBS and resuspended in annexin-V-FluosLUOS labeling solution 100 μL. The cells incubated at 25°C for 15 min were subjected to flow cytometric analysis following the manufacturer’s instructions (BD FACSCalibur).

### Western blotting

Total protein was extracted from cells using lysis buffer and its concentration was detected with the Pierce BCA Protein Assay Kit. Equal amounts of proteins (30–100 μg/sample) were resolved on 12% polyacrylamide-SDS gels and transferred to polyvinylidene difluoride membranes blocked with 5% w/v nonfat dry milk (Millipore, MA, USA). Membranes were incubated with primary antibodies at 4°C overnight. The membranes washed with TBS-T were incubated with horseradish peroxidase-labeled secondary antibodies (Proteintech, Chicago, IL, USA) for 1.5 h at 25°C. Immunobands were visualized using an enhanced chemiluminescence kit (GE Healthcare, Waukesha, WI, USA). An imaging densitometer was used to scan the protein bands. β-Actin was used as a loading control.

### Statistical analysis

All statistical analyses were performed using SAS ver. 9.2 (SAS Institute, Cary, NC, USA). All data were expressed as mean ± SD deviation of triplicate experiments. Comparisons were made using a one-way ANOVA followed by Dunnett's test. *P* < 0.05 was considered as a significant. *P* < 0.001 was considered as a highly significant difference.

## Results

### SA and SB prevent the loss of cell viability by UVB irradiation in HaCaT cells

**Fig 1:**
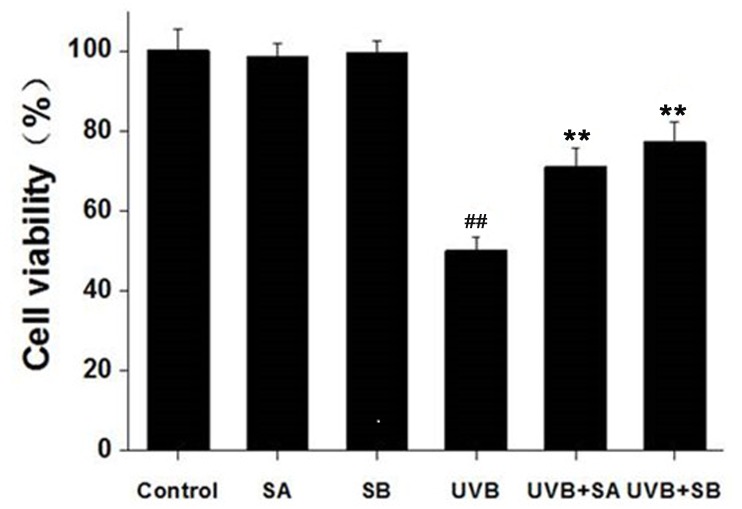
SA and SB reduced the loss of cell viability of HaCaT cells after UVB exposure. HaCaT cells were treated with 100 μM SA or SB for 24 h, followed by exposure to 30 mJ/cm^2^ of UVB. Cell viability was evaluated by using the MTT assay for 24 h from UVB exposure. ##*P* < 0.001 was considered as a highly significant difference compared with the control group. **P < 0.001 was considered as a highly significant difference compared with the UVB-irradiated only group.

To assess the effects of SA and SB on cell viability following UVB irradiation, we treated HaCaT cells with 100 μM SA or SB for 24 h, followed by exposure to 30 mJ/cm^2^ of UVB. Cell viability was measured by using the MTT assay at 24 h after UVB irradiation. UVB significantly reduced cell viability (Fig 1). Treatment with either SA or SB alone did not alter cell viability, whereas pretreatment with either SA or SB significantly protected the cells from UVB-induced cell death. These results indicate that SA and SB can protect HaCaT cells after UVB irradiation.

### SA and SB effectively scavenge UVB-induced ROS in HaCaT cells

**Fig 2:**
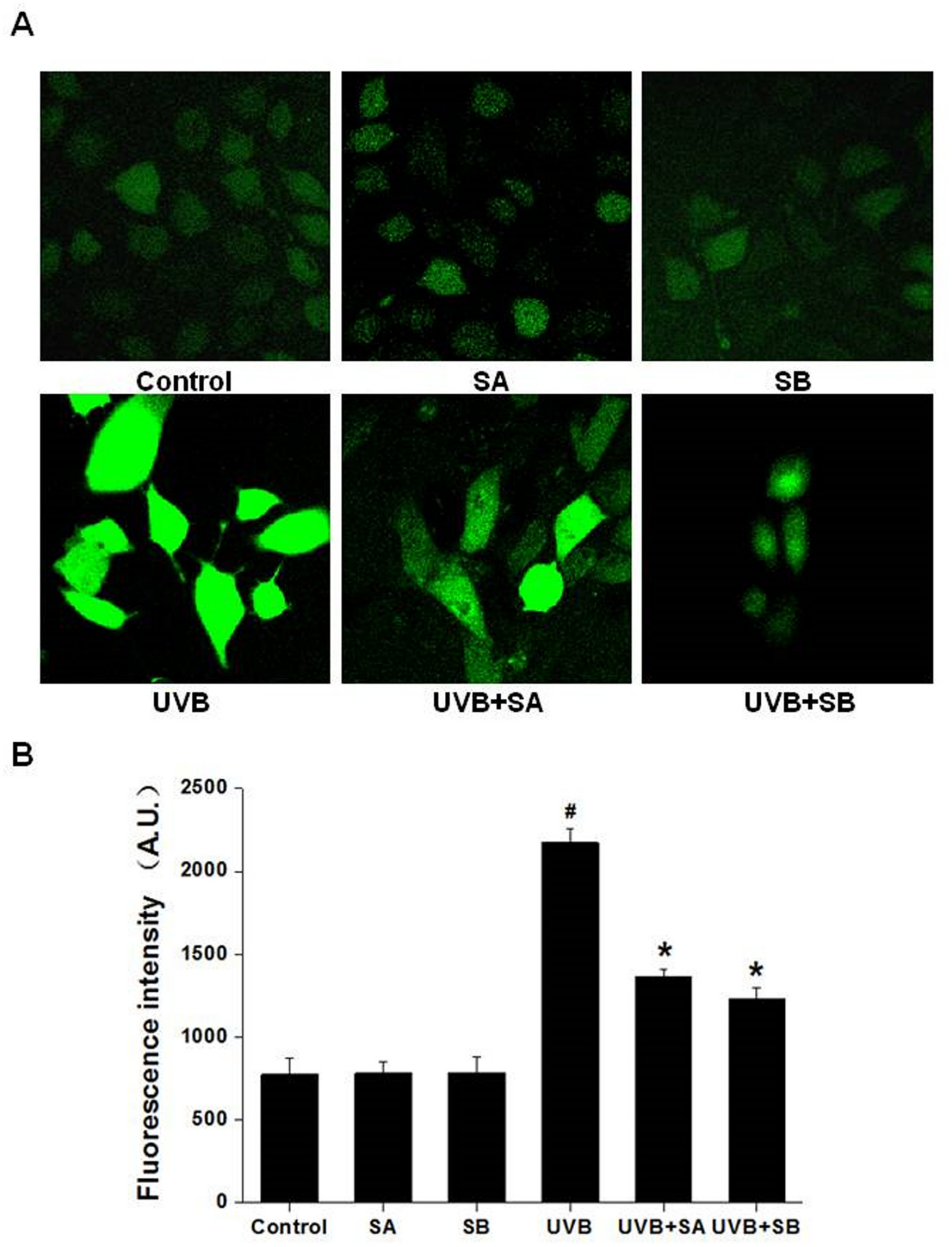
SA and SB effectively prevented UVB-produced ROS in HaCaT cells. HaCaT cells were treated with 100 μM SA or SB for 24 h, followed by exposure to 30 mJ/cm^2^ of UVB. The levels of ROS were measured by the DCFH-DA assay. (A) Representative laser scanning confocal microscopy images, 400 × magnification. (B) Quantification of the fluorescence intensities of the images in panel A. #*P* < 0.05 was considered as a significant difference compared with the control group. *P < 0.05 was considered as a significant difference compared with the only UVB-irradiated group.

To investigate the effects of SA and SB on the level of UVB-induced intracellular ROS, we cultured HaCaT cells with SA or SB followed by UVB exposure, and the ROS level was measured by the DCFH-DA assay (Fig 2). In comparison to the untreated control, SA or SB alone (without UVB-irradiation) had no effect on the generation of ROS. In sharp contrast, the ROS level was dramatically increased in the UVB-irradiated cells (Fig 2A). Interestingly, the ROS levels were significantly reduced in cells pretreated with SA or SB followed by UVB exposure Fig (2A–2B). These results demonstrate that SA and SB can effectively scavenge ROS induced by UVB.

### SA and SB reduce UVB-induced DNA damage in HaCaT cells

**Fig 3:**
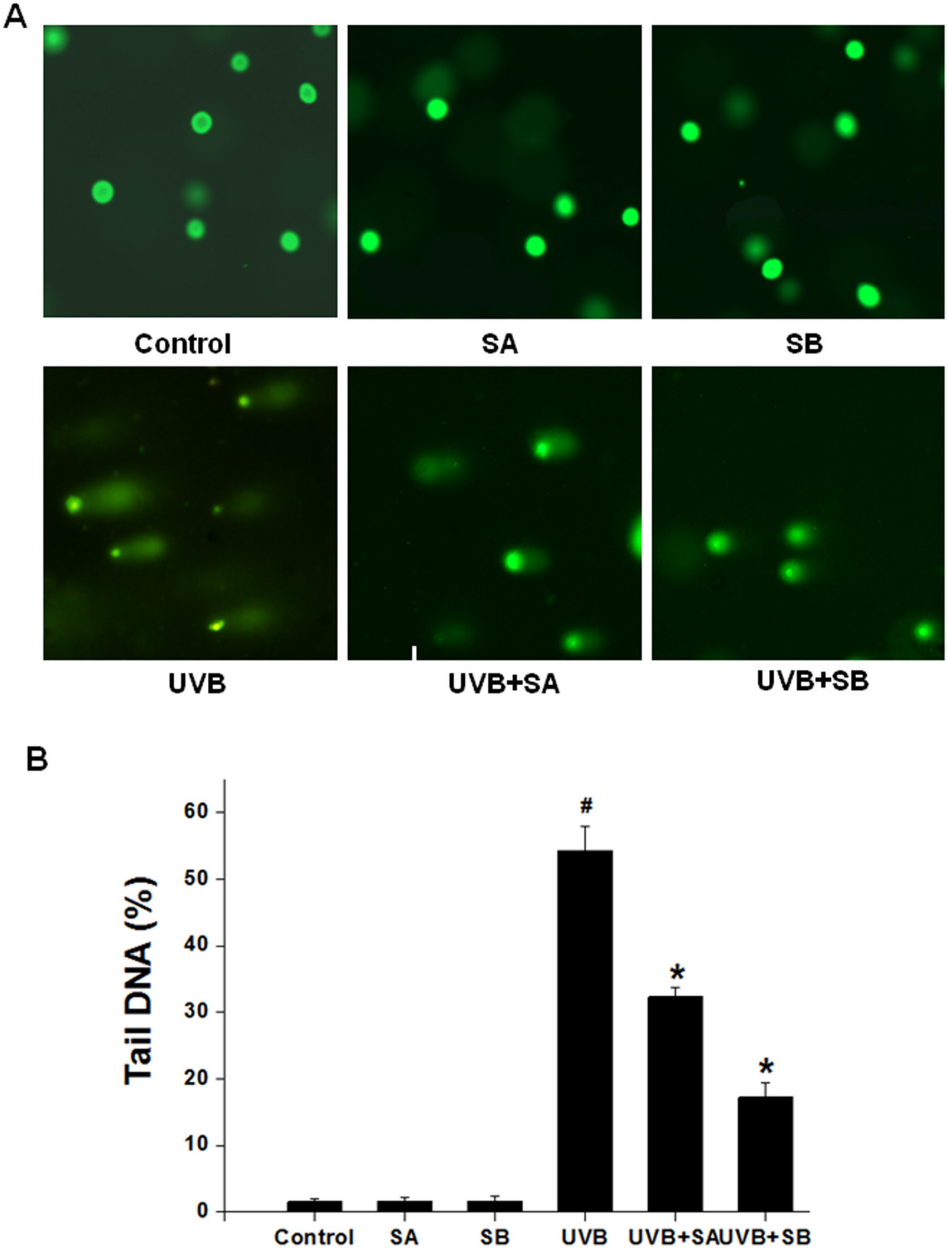
SA and SB reduced the DNA damage that was induced by UVB in HaCaT cells. HaCaT cells were treated with 100 μM SA or SB for 24 h, followed by exposure to 30 mJ/cm^2^ of UVB. DNA damage was assessed using a comet assay at 24 h after UVB treatment. The percent of tail DNA was analyzed. (A) Representative epifluorescence microscopy images, 200× magnification. (B) Quantification of the fluorescence intensities of the images in panel A. #*P* < 0.05 was considered as a significant difference compared with the control group. *P < 0.05 was considered as a significant difference compared with the only UVB-irradiated group.

UV radiation has been shown to produce DNA damage directly and indirectly. To test whether SA and SB protect cells from UVB-induced DNA damage, we measured the DNA damage of HaCaT cells exposed to UVB irradiation in the presence or absence of SA or SB by the comet assay. Damaged cellular DNA is separated from intact DNA, yielding a classic “comet tail” shape under a microscope. The percentage of tail DNA is a commonly used parameter to analyze comet assay results by means of CASP software programs. A total of 50–100 cells were analyzed per sample. As shown in Fig 3A and 3B, SA or SB treatment alone did not result in a “comet tail”. In contrast, UVB irradiation led to a long “comet tail”, indicating extensive DNA strand breaks. However, the degree of DNA damage was apparently reduced in the cells pretreated with SA or SB followed by UVB treatment. These results demonstrate that SA and SB may reduce DNA damage by reducing ROS indirectly.

### SA and SB have an inhibitory effect on UVB-induced cell apoptosis in HaCaT cells

**Fig 4:**
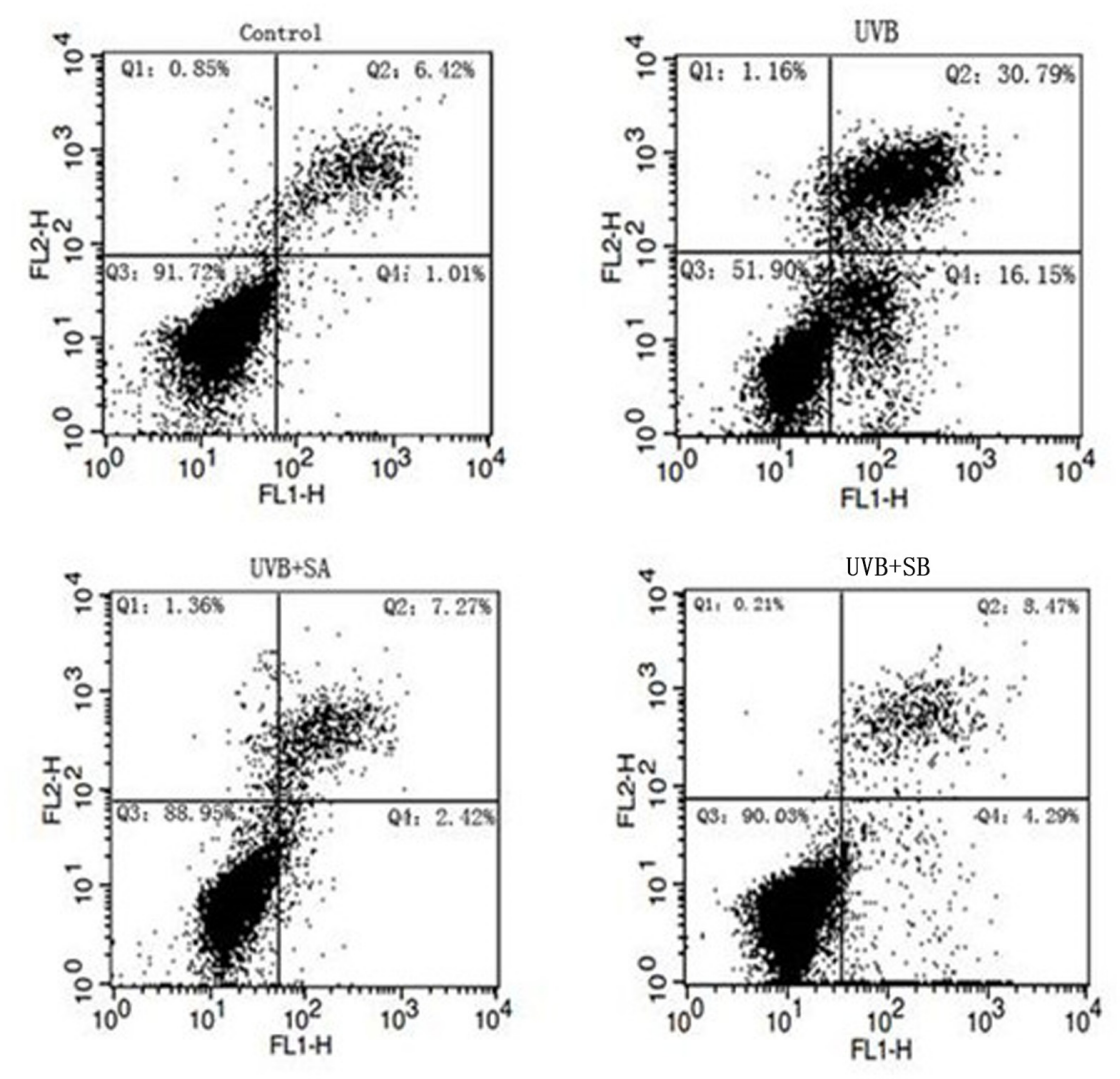
SA and SB protected against UVB-induced cell apoptosis in HaCaT cells. HaCaT cells were treated with 100 μM SA or SB for 24 h, followed by exposure to 30 mJ/cm^2^ of UVB. After 24h, the apoptotic cells were dyed with Annexin-V-Fluos (Q4) and analyzed using a flow cytometry assay. Control Q4: 1.01%. UVB Q4: 16.15%. UVB+SA Q4: 2.42%. UVB+SB Q4: 4.29%.

Either ROS or DNA damage can induce the activation of the DNA damage response, which activates a series of signaling pathways to promote cell apoptosis. Protection against the UVB-induced production of ROS and DNA damage by SA or SB suggests that SA and SB may prevent cell apoptosis after UVB irradiation. To test this hypothesis, we measured the cell apoptosis of HaCaT cells exposed to UVB in the presence or absence of SA or SB by the Annexin-V-Fluos Staining kit coupled with flow cytometric analyses. UVB irradiation increased the percentage of apoptotic cells from 1.01% to 16.15%, which was significantly reduced to 2.42% by SA and 4.29% by SB (Fig 4). These results demonstrate that SA and SB have inhibitory effects on UVB-induced cell apoptosis probably by reducing DNA damage and ROS.

### SA and SB prevent UVB-mediated cleavage of caspase-3, caspase-8 and caspase-9 in HaCaT cells

**Fig 5:**
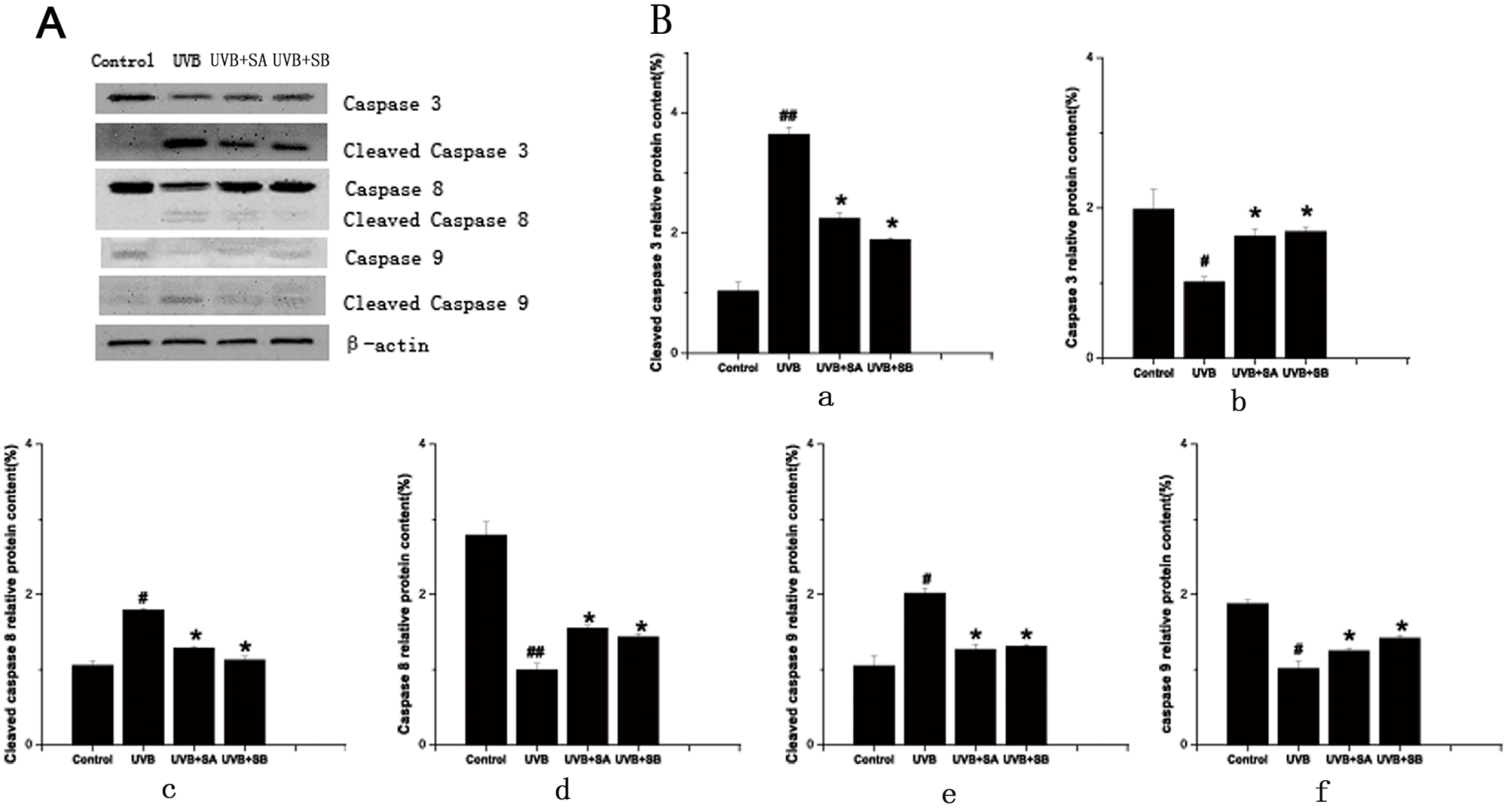
SA and SB abolished the UVB-mediated cleavage of caspase-3, caspase-8 and caspase-9 levels in HaCaT cells. HaCaT cells were treated with 100 μM SA or SB for 24 h, followed by exposure to 30 mJ/cm^2^ of UVB. After 24 h, the cells were performed. (A) The expression of caspase-3, cleaved caspase-3, caspase-8, cleaved caspase-8, caspase-9 and cleaved caspase-9 were determined using western blots, with β-actin as the loading control. (B) The relative protein content of the bands was normalized to β-actin. a: Cleaved caspase-3 relative protein content(%); b: Caspase-3 relative protein content(%); c: Cleaved caspase-8 relative protein content(%); d: Caspase-8 relative protein content(%); e: Cleaved caspase-9 relative protein content(%); f: Caspase-9 relative protein content(%).

It has been reported that UVB irradiation induces apoptotic cell death in HaCaT cells via activation of caspase-3, caspase-8 and caspase-9 [21]. To further support our observation that SA and SB protected HaCaT cells from apoptosis after UVB exposure, the expression of caspase-3 caspase-8 and caspase-9 were determined in HaCaT cells exposed to UVB in the presence or absence of SA or SB by western blot. Compared with the untreated control cells, UVB irradiation apparently led to a decrease of caspase-3, caspase-8 and caspase-9, and an increase of cleaved caspase-3, cleaved caspase-8 and cleaved caspase-9, indicating the activation of apoptosis. In contrast, pretreatment with SA or SB obviously reduced the decrease of caspase-3 caspase-8 and caspase-9 levels and the increase of cleaved caspase-3, cleaved caspase-8 and cleaved caspase-9 levels after UVB exposure (Fig 5), indicating that SA and SB could protect against UVB-induced activation of caspases.

## Discussion

In our study, the protective effects of SA and SB against UVB-induced damage were investigated in HaCaT cells. Our results showed that SA and SB apparently prevented the UVB-induced loss of cell viability and production of ROS. Moreover, SA and SB decreased the UVB-induced DNA damage in HaCaT cells. Accordingly, SA and SB effectively prevented UVB-induced cell apoptosis in HaCaT cells. It is implied that the antioxidants SA and SB could protect cells against UVB-induced cell damage by scavenging ROS.

*Schisandra chinensis (Turcz*.*)Baill* grows mainly in China, Japan, and Korea. The seeds and fruits have been used as medicines for centuries [11]. *S*. *chinensis* also exhibits strong antioxidant activities in different levels, such as isolated organs, tissues, cells, and enzymes [22]. SA and SB, as the major lignans isolated from *S*. *chinensis*, demonstrate high antioxidant potential both *in vitro* and *in vivo* and have been found to exhibit beneficial bioactivities such as antihepatotoxicity and antioxidation [14,23]. In this study, we provided evidence that SA and SB could prevent against UVB-induced ROS production.

UVB radiation critically damages cellular macromolecules and induces ROS, which activate signaling pathways such as inducing oxidative damage to DNA and cellular proteins [6]. UVB light, which consists of wavelengths common in solar radiation, corresponds to the most cytotoxic and mutagenic wavelengths. DNA directly absorbs incident photons within this narrow wavelength range. ROS can also damage DNA indirectly [4]. In our study, *in vitro* results indicated that SA and SB have the potential efficacy to ameliorate UVB-induced skin damage. However, it remains to be determined whether SA and SB effectively suppress DNA damage by reducing ROS production indirectly or stop DNA from absorbing incident photons directly.

In addition, UV radiation induces cell apoptosis via generation of ROS, which can change mitochondrial transmembrane protein, promote the release of the cytochrome C and apoptosis inducing factor (AIF), and activate caspases [19, 20, 24]. Caspases play major roles in cell apoptosis and Caspase cleavage cascade begins with initiator Caspase activation. Caspase 9, Caspase 8 are initiator caspases and Caspase 3 is an effector caspase[25, 26]. In this study, we found that caspase-3, caspase-8 and caspase-9 were activated after UVB treatment, which were abolished by SA or SB pretreatment. Our results suggest that SA and SB may prevent keratinocytes from apoptotic death after UVB exposure by scavenging ROS, increasing the mitochondrial membrane protein activation, inhibiting caspase cleavage.

In summary, we demonstrated that the antioxidants SA and SB effectively protected against UVB-induced cell death by scavenging ROS and reducing DNA damage in HaCaT cells. Our findings suggested that the natural plant ingredients SA and SB may be developed as agents that protect against ultraviolet radiation as well as in natural cosmetics.
